# Supplementary material for: Cloud BioLinux: pre-configured and on-demand bioinformatics computing for the genomics community
Source: BMC Bioinformatics. 2012 Mar 19;13:42. doi: 10.1186/1471-2105-13-42 (PMC3372431; doi:10.1186/1471-2105-13-42)
Supplement: Additional file 1 — Supplementary 1 Cloud BioLinux software documentation in the form of a mini, self-contained website. Users need to download and uncompress the .zip file, and open through a web browser the "index.html" file available on the main directory. (ZIP 1823 kb). [file 1471-2105-13-42-S1.ZIP › Cloud-BioLinux-Package-Documentation/docs/consed.html]

Bio-Linux Software Documentation Pages

Back to search form

## consed

|  |  |
| --- | --- |
| Name | consed |
| Description | **Consed/Autofinish** is a tool for viewing, editing, and finishing sequence assemblies. Consed can handle data from Sanger, 454 and Solexa/Illumina technologies. Finishing capabilities include allowing the user to pick primers and templates, suggesting additional sequencing reactions to perform, and facilitating checking the accuracy of the assembly using digest and forward/reverse pair information.  One difference from the standard Consed distribution that users should be aware of is that the sffinfo binary distributed with Consed is called using the command: `sffinfo_consed` if you have installed bio-linux-consed. This is to avoid conflict with other binaries with the name sffinfo. Check out the readme.biolinux file for other information specific to the bio-linux-consed pacakage.  The Consed software comes with a number of scripts including phredPhrap. To run phredPhrap, type **phredPhrap** from within your edit\_dir directory. To run phredPhrap using polyphred, type the command **phredPhrap polyphred**. **Important:** The Bio-Linux consed package assumes you have named your traces according to the St. Louis naming scheme. If you have not used this naming scheme you **must** refer to the documentation to address this!  The link to the README.txt file below will only be active if you have already installed the Bio-Linux consed package.  **Consed** is ***licensed software*** and thus is ***not*** installed by default on Bio-Linux. It is free to academic researchers but you must obtain a license. Further information on how to obtain consed is available on the official website for phred, phrap and consed.  The Bio-Linux consed package depends on the Bio-Linux packages for phrap and phd2fasta. Thus, to obtain and install bio-linux-consed, you must have a license for phrap must obtain and install the bio-linux-phrap package from us before you attempt to install bio-linux-consed. |
| Homepage | http://www.phrap.org |
| Remote Documentation | http://www.phrap.org/phredphrapconsed.html#block\_consed   http://bozeman.mbt.washington.edu/consed/consed.html |
